# Large angular correction of arithmetic coronal alignment is associated with residual lateral laxity after total knee arthroplasty in varus knees for Japanese patients

**DOI:** 10.1002/jeo2.12100

**Published:** 2024-08-13

**Authors:** Junya Itou, Umito Kuwashima, Masafumi Itoh, Ken Okazaki

**Affiliations:** ^1^ Department of Orthopaedic Surgery Tokyo Women's Medical University Tokyo Japan

**Keywords:** alignment, coronal plane alignment of the knee, patient‐reported outcome measures, total knee arthroplasty, varus

## Abstract

**Purpose:**

One of the most common types of coronal plane alignment of the knee (CPAK) is type I, which is characterised by varus alignment and apex distal joint line obliquity. The purpose of this study was to evaluate the association of changes in arithmetic hip–knee–ankle angle (aHKA) with both postoperative joint laxity and patient‐reported outcome measures (PROMs) in patients with CPAK type I following mechanical alignment (MA) total knee arthroplasty (TKA).

**Methods:**

Of 111 consecutive knees in 92 patients with osteoarthritis who underwent primary TKA, 80 knees (72.0%) with CPAK type I phenotype preoperatively were evaluated. All TKAs were performed to achieve neutral MA by a medial stabilising gap balancing technique. Pre‐ to postoperative change in aHKA was defined as ΔaHKA. The 80 CPAK type I knees were divided into a larger ΔaHKA group (>7°) and a smaller ΔaHKA group (≤7°). PROMs, including the Knee Society Score and Forgotten Joint Score‐12, were assessed before and 2 years after surgery. Pre‐ and postoperative joint laxity was assessed using a Telos arthrometer.

**Results:**

Twenty‐two knees showed a larger ΔaHKA, and postoperative lateral joint laxity in varus stress was significantly greater in these patients than in those with a smaller ΔaHKA (6.8° vs. 4.5°, *p* = 0.006). There were no significant differences between the groups in PROMs (*p* = n.s.).

**Conclusions:**

Postoperative lateral laxity was associated with larger aHKA changes than smaller aHKA changes in CPAK type I knees after TKA. However, no statistically significant differences in PROMs were found according to the amount of change in aHKA.

**Level of Evidence:**

Level III.

**Abbreviations**
aHKA: arithmetic hip–knee–ankle angle
CPAK: coronal plane alignment of the knee
FJS‐12: Forgotten Joint Score
HKA: hip–knee–ankle angle
JLCA: joint line convergence angle
JLO: joint line obliquity
KA: kinematic alignment
KSS: Knee Society Score 2011
K/L: Kellgren–Lawrence
MA: mechanical alignment
mLDFA: mechanical lateral distal femoral angle
MPTA: medial proximal tibial angle
TKA: total knee arthroplasty

## INTRODUCTION

Several competing alignment philosophies have recently been introduced to improve patient satisfaction following total knee arthroplasties (TKAs) [1, 22, 31]. While mechanical alignment (MA) remains the benchmark, Howell et al. have introduced kinematic alignment (KA) [10]. KA aims to restore the constitutional prearthritic knee joint, and good clinical outcomes of TKA have been reported [5, 22].

MacDessi et al. recently introduced the coronal plane alignment of the knee (CPAK) system for classifying knee phenotypes independent of changes in osteoarthritic alignment [19]. When optimising soft tissue balance, the CPAK classification is useful when considering which knee would be suitable for KA during TKA. Especially in CPAK type I characterised by varus alignment and apex distal joint line obliquity (JLO), it has been reported that soft tissue balance and clinical outcomes following MA TKA are inferior to those following KA TKA [2]. However, knees with the same CPAK type I classification require different amounts of coronal plane alignment correction to achieve MA. In recent studies from Asia, it was found that more than 50% of the patients had CPAK type I knees [28, 33] and that this proportion was likely to differ by ethnicity [28]. It is well known that the most prevalent angular deformity in the coronal plane is varus deformity of the knee, and how to treat the varus knee is of interest to many surgeons [6, 8, 31]. Furthermore, there is considerable variability in the arithmetic hip–knee–ankle angle (aHKA) in Asian cohorts, which often include knees with severe varus, even in those that are classified as having the same CPAK type I phenotype. Hence, in the case of MA TKA, the clinical benefit of surgery may vary depending on the degree of preoperative constitutional varus.

Large angular correction of aHKA in varus knees would, however, result in soft tissue imbalance with significant lateral laxity, and knees with severe varus deformity are sometimes associated with elongated lateral soft tissues, which would worsen the soft tissue balance further [3, 9, 26]. Therefore, a milder angular correction that achieves a slightly varus alignment would be a better target for knees with severe varus. Concepts of restricted KA or adjusted MA [22] would be consistent with this concept. Although some studies have shown that patients with varus knees have better clinical outcomes if they have a slightly varus alignment after MA TKA compared with those who have neutral alignment, they did not account for the size of the correction angle [24, 30]. Even if the final alignment after TKA is the same, the ligament balance and clinical outcomes would depend on the correction angle required to achieve alignment.

The purpose of this study was to evaluate the association between changes in aHKA and both postoperative joint laxity and patient‐reported outcome measures (PROMs) in patients with CPAK type I following MA TKA. It was hypothesised that a larger postoperative change in aHKA would result in increased lateral laxity and worse PROMs in patients with the CPAK type I phenotype. These findings would be helpful when considering the rationale for alternative coronal alignment, such as KA or restricted KA.

## MATERIALS AND METHODS

**Figure 1:**
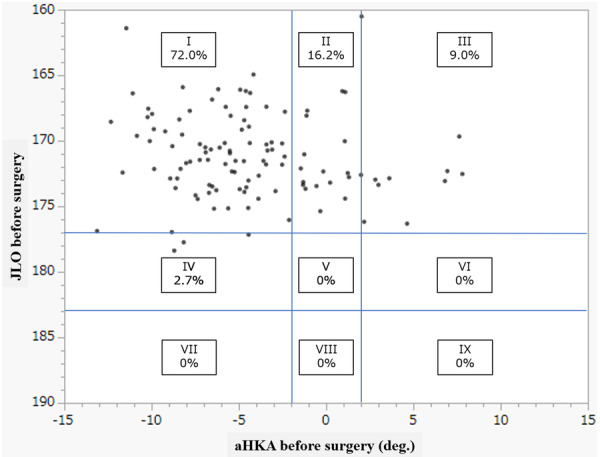
Distribution of coronal plane alignment of the knee (CPAK) types before surgery in all patients. aHKA, arithmetic hip–knee–ankle angle; JLO, joint line obliquity.

This retrospective study included 111 consecutive knees from 92 patients who underwent primary TKA performed using the Journey II system (Smith & Nephew) for osteoarthritis between April 2017 and March 2021 with a minimum of 2 years of follow‐up. Routinely, each TKA patient completed a series of PROMs and had full‐length weight‐bearing radiographs taken. Preoperative CPAK classification is shown in Figure 1: type I was most common (72.0%), followed by type II (16.2%), type III (9.0%) and type IV (2.7%). There were no cases of types V–IX. The inclusion criterion was having a knee with the CPAK type I phenotype preoperatively. The exclusion criteria were additional surgery on the index knee for infection or aseptic loosening during the follow‐up period. Finally, 80 primary TKAs in 69 patients (72.0%) who met the study criteria were included in the study. Patient data, including age, sex and preoperative body mass index, were collected from the medical records.

The study protocol was approved by our institutional ethics committee (approval number 4952). The study was conducted in accordance with the ethical standards laid down in the 1964 Declaration of Helsinki and its later amendments. Informed consent was obtained via the opt‐out route.

### Radiological parameters and CPAK classification

The radiological measurements were obtained using whole‐leg standing radiographs in accordance with the procedure described by MacDessi et al. [19, 20] and included the mechanical lateral distal femur angle (mLDFA) and mechanical medial proximal tibia angle (MPTA). For the postoperative MPTA measurement, the tangent line of the articular plane was defined as the joint line of the distal femur, not the tibial base plate, considering the insert geometry of the Journey II [27]. The aHKA and JLO were calculated as follows: aHKA = MPTA − mLDFA; JLO = MPTA + mLDFA. Nine subgroups were included in the CPAK classification and categorised as neutral (aHKA 0° ± 2°), valgus (aHKA > 2°), varus (aHKA < 2°), aHKA and neutral (JLO 180° ± 3°), apex proximal (JLO < 177°) or apex distal JLO (JLO > 183°).

The postoperative change in aHKA was defined as the ΔaHKA. Similarly, the change in aHKA and JLO postoperatively was defined as ‘distance’; that is, how far the knee was corrected from before to after TKA in the CPAK coordinate system. The formula for determining ‘distance’ was as follows: distance = $\surd ({\Delta \mathrm{aHKA}}^{2}+{\Delta \mathrm{JLO}}^{2})$.

**Figure 2:**
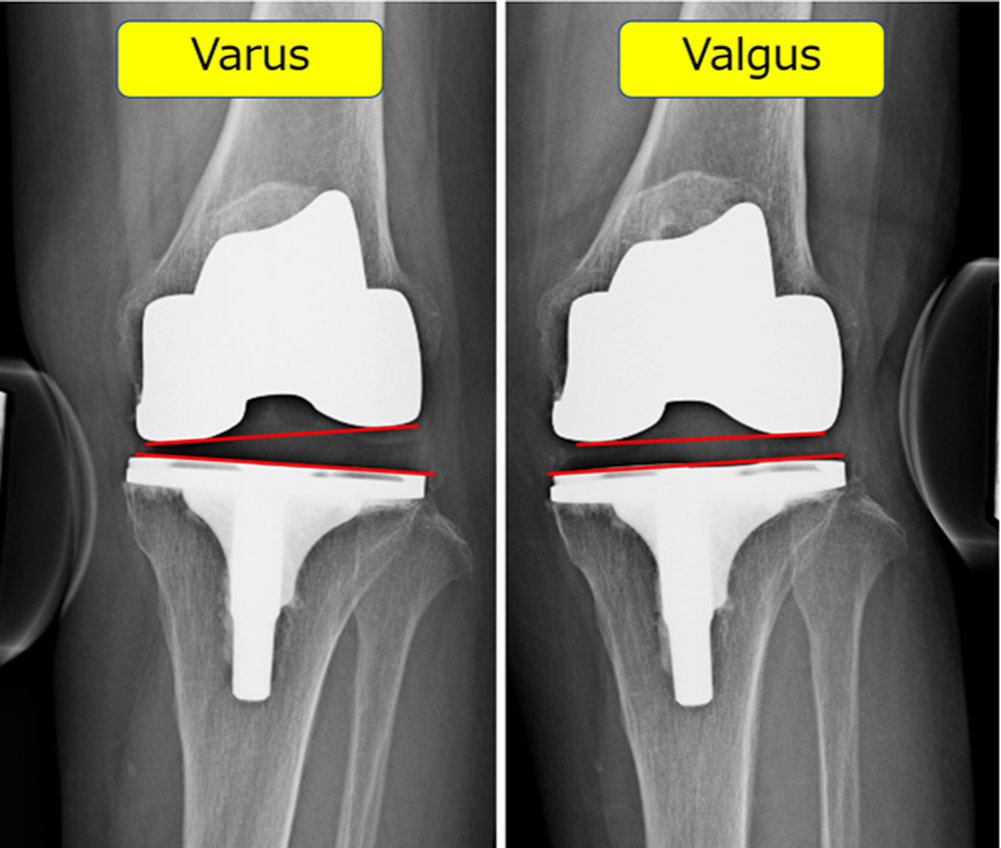
Stress radiographs after total knee arthroplasty. Varus and valgus stress radiographs were used to evaluate the stability of the knee during extension. The stress angle measured was deemed to be positive if the lateral gap was greater than the medial gap. Three degrees were subtracted from the angles measured.

Postoperative varus and valgus stability of the knee joint in extension was evaluated using a Telos arthrometer (SD 900 Stress Device, Telos Medical Co. Ltd.) [14]. Postoperative stress radiographs were obtained at 12 months after TKA. A 150‐N load was applied just above the joint on the medial or lateral femoral condyle during the varus and valgus stress test. The stress angle was defined as the angle between the line through the distal convex margin of the femoral condyles and the tibial boundary and was deemed to be positive if the lateral gap was greater than the medial gap (Figure 2). Postoperatively, the measurements were subtracted by 3° to account for the inclination of the polyethylene insert in Journey II [27]. The angle was deemed to be positive if the lateral gap was greater than the medial gap.

Two independent blinded observers (J. I., U. K.) assessed the aHKA for each of the 20 randomly selected patients and intra‐ and interobserver reliability was evaluated.

### Surgical technique and rehabilitation

One of the four experienced knee surgeons performed all the operations using a tourniquet and a subvastus approach. All TKAs were performed using the gap‐balancing technique to achieve medial knee stability throughout the range of motion in neutral MA with standard surgical instruments [11, 13]. A tibial osteotomy was performed perpendicular to the mechanical axis of the tibia in the coronal plane and with 3° of posterior inclination along the sagittal plane using an extramedullary resection guide. A distal femoral osteotomy was performed perpendicular to the mechanical axis of the femur utilising an intramedullary resection guide. Femoral external rotation was set 3° to the posterior condylar axis, while the anterior–posterior position of the femoral component was adjusted to obtain the same gap lengths between extension and flexion on the medial side [17, 27]. No medial soft tissue release was performed to obtain medial stability, and residual lateral laxity was accepted. All components were cemented. The choice of a cruciate‐retaining insert or bicruciate stabilised insert was left to the surgeon's discretion.

Patients were allowed to walk while fully weight‐bearing from the first postoperative day and underwent rehabilitation without restriction of range of motion.

### PROMs

Patients were asked by the attending surgeon to complete the Knee Society Score 2011 (KSS) and Forgotten Joint Score‐12 (FJS‐12) questionnaires preoperatively and 2 years postoperatively for evaluation of PROMs [25].

### Statistical analysis

Descriptive statistics are reported as the median (range), number (percentage) or mean and standard deviation. The distribution of continuous variables was assessed for normality by visual inspection of histograms and the Shapiro–Wilk test. Differences between the two alignment groups were examined using the *χ* ^2^ test for categorical variables and the Wilcoxon signed‐rank test for continuous variables. Spearman's rank correlation analysis was performed to assess the correlations between distance or ΔaHKA and stability parameters (varus and valgus angles in extension) and each PROM. As in a previous study [15], the intraclass correlation coefficient was rated as poor (<0.5), moderate (0.5–0.75), good (0.75–0.9) or excellent (>0.9).

A post hoc power analysis was conducted using G*Power (version 3.1.9.7). Based on an effect size of 0.5, a total sample size of 116 and an *⍺* error probability of 0.05 for two groups, it was calculated that a power of 0.83 would be required. All statistical analyses were performed using JMP software version 17 (SAS Institute Inc.). A *p* < 0.05 was considered statistically significant.

## RESULTS

**Figure 3:**
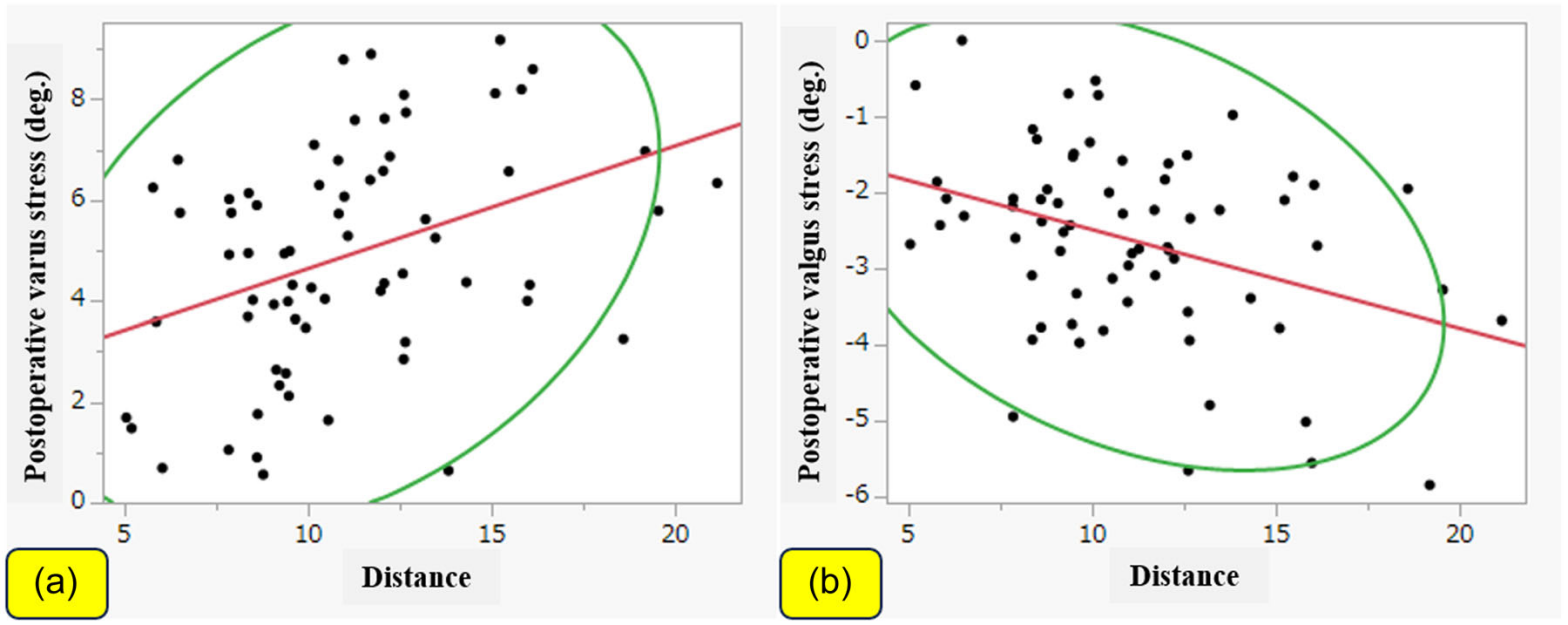
Correlations of distance with postoperative stress angles. Distance was weakly correlated with the postoperative varus and valgus stress angles. (a) Varus stress (|*r*| = 0.37, *p* = 0.001); (b) valgus stress (|*r*| = 0.36, *p* = 0.002).

**Figure 4:**
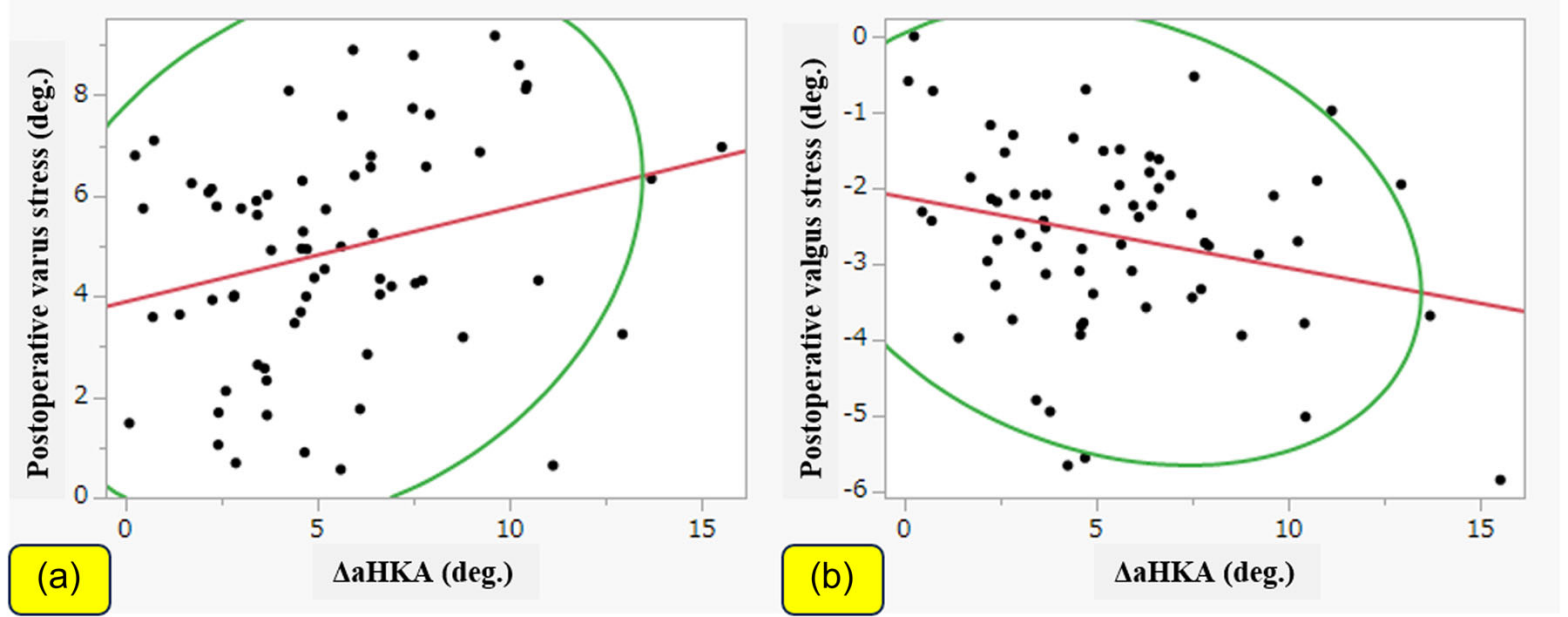
Correlations of postoperative change in arithmetic hip–knee–ankle angle (ΔaHKA) with postoperative stress angles. The ΔaHKA was weakly correlated with the postoperative varus and valgus stress angles. (a) Varus stress (|*r*| = 0.27, *p* = 0.02); (b) valgus stress (|*r*| = 0.24, *p* = 0.04).

Patient demographic and radiological data are shown in Table 1. Distance was weakly correlated with the postoperative varus and valgus stress angles (|*r*| = 0.37, *p* = 0.001 and |*r*| = 0.36, *p* = 0.002, respectively; Figure 3). On the other hand, there were no significant correlations between distance and preoperative varus or valgus stress. Similarly, the ΔaHKA was weakly correlated with the postoperative varus and valgus stress angles (|*r*| = 0.27, *p* = 0.02 and |*r*| = 0.24, *p* = 0.04, respectively; Figure 4). There were no significant correlations between the ΔaHKA and preoperative varus or valgus stress. There were also no significant correlations between distance or ΔaHKA and any of the PROMs.

**Table 1:** Demographic and radiological data for preoperative CPAK type I.

Parameter	Value
Age at surgery, years, median [range]	73.5 [50–85]
Male sex, *n* (%)	20 (25.0)
Body mass index, median [range]	25.6 [20.5–35.2]
Operated side: right (%)	46 (57.5)
Mechanism, *n* (%)	BCS, 64 (80.0); CR, 16 (20.0)
Preoperative ROM (°), [range]	117.5 [55–145]
Postoperative ROM (°), [range]	130 [75–145]
Follow‐up, months, median [range]	24.0 [24–60]
Preoperative MPTA (°), mean ± SD	82.1 ± 2.1
Postoperative MPTA (°), mean ± SD	86.6 ± 1.7
Preoperative mLDFA (°), mean ± SD	88.5 ± 1.8
Postoperative mLDFA (°), mean ± SD	87.6 ± 1.8
Preoperative aHKA (°), mean ± SD	−6.4 ± 2.6
Postoperative aHKA (°), mean ± SD	−0.9 ± 2.4
Preoperative JLO (°), mean ± SD	170.7 ± 2.9
Postoperative JLO (°), mean ± SD	174.3 ± 2.5

*Note*: °, degree.

Abbreviations: aHKA, arithmetic hip–knee–ankle angle; BCS, bicruciate stabilised; CPAK coronal plane alignment of the knee; CR, cruciate‐retaining; JLO, joint line obliquity; mLDFA, mechanical lateral distal femoral angle; MPTA, medial proximal tibial angle; ROM, range of motion; SD, standard deviation.

Considering that the mean preoperative aHKA was −6.4° in CPAK type I, the knees were divided into a larger ΔaHKA group (>7°) and a smaller ΔaHKA group (≤7°). There were 22 knees in the larger ΔaHKA group (>7°) (Table 2). Postoperative lateral joint laxity in varus stress was significantly greater in the larger ΔaHKA group than in the smaller ΔaHKA group (6.8° vs. 4.5°, *p* = 0.006).

**Table 2:** Differences in demographic and radiological data for preoperative CPAK type I by correction angle size.

Parameter	Larger ΔaHKA group (>7°) (*n *= 22)	Smaller ΔaHKA group (≤7°) (*n *= 58)	*p* Value
Age at surgery, years, median [range]	76 [63–84]	73 [50–85]	0.17
Male sex, *n* (%)	6 (27.3)	14 (24.1)	0.77
Body mass index, median [range]	26.5 [20.5–35.1]	25.5 [20.5–35.2]	0.90
Preoperative ROM (°), [range]	110 [55–135]	120 [65–145]	0.14
Postoperative ROM (°), [range]	130 [95–145]	130 [75–145]	0.69
ΔaHKA (°), mean ± SD	9.6 ± 2.3	3.9 ± 1.8	**<0.0001**
ΔJLO (°), mean ± SD	3.7 ± 273	4.1 ± 2.9	0.59
Distance, mean ± SD	14.1 ± 3.0	9.8 ± 2.8	**<0.001**
Preoperative stress (varus) (°), median [range]	6.7 [2.5–12.2]	6.5 [0.3–11.9]	0.47
Preoperative stress (valgus) (°), median [range]	−0.2 [−4.6, 2.8]	0.6 [−8.5–4.4]	0.62
Postoperative stress (varus) (°), median [range]	6.8 [0.6–9.2]	4.5 [0.6–8.9]	**0.006**
Postoperative stress (valgus) (°), median [range]	−2.8 [−5.8, −0.5]	−2.3 [−5.6, 0]	0.19

*Note*: Bold values denote statistically significant differences between the alignment groups. ˚, degree.

Abbreviations: ΔaHKA, change in arithmetic hip–knee–ankle angle; CPAK, coronal plane alignment of the knee; JLO, joint line obliquity; K/L, Kellgren–Lawrence; ROM, range of motion; SD, standard deviation.

There was no significant difference between the two groups in any of the PROMs (Table 3). Improvements in the FJS‐12, KSS satisfaction and KSS function scores were greater in the smaller ΔaHKA group than in the larger ΔaHKA group, but the differences were not statistically significant. Intraobserver reliability was 0.97 and interobserver reliability was 0.87.

**Table 3:** Comparison of patient‐reported outcome measures for CPAK type I knees.

PROMs	Larger ΔaHKA group (>7°) (*n *= 22)	Smaller ΔaHKA group (≤7°) (*n *= 22)	Mean difference (95% CI)	*p* Value
Preoperative				
FJS‐12	19.9 ± 14.9	19.1 ± 14.7	0.8 (−6.6, 8.2)	0.81
KSS symptoms	8.1 ± 5.2	7.3 ± 4.9	0.7 (−1.7, 3.3)	0.53
KSS satisfaction	15.2 ± 6.1	14.5 ± 7.3	0.7 (−2.7, 4.2)	0.55
KSS function	50.9 ± 20.9	46.7 ± 16.8	4.1 (−4.8, 13.1)	0.41
KSS total	88.2 ± 27.3	82.5 ± 23.6	5.6 (−6.6, 17.9)	0.41
Postoperative				
FJS‐12	54.2 ± 28.4	59.3 ± 23.1	5.1 (−7.2, 17.4)	0.42
KSS symptoms	22.2 ± 2.9	21.3 ± 4.3	0.9 (−1.1, 2.9)	0.65
KSS satisfaction	28.9 ± 8.1	30.8 ± 6.9	1.9 (−1.6, 5.6)	0.29
KSS function	75.2 ± 19.3	76.2 ± 16.4	1.0 (−7.8, 9.9)	0.81
KSS total	137.1 ± 26.1	137.7 ± 25.1	0.5 (−12.5, 13.6)	0.93
Improvement				
ΔFJS‐12	34.3 ± 28.3	40.2 ± 24.4	5.9 (−6.8, 18.6)	0.23
ΔKSS symptoms	14.1 ± 6.2	13.9 ± 6.6	0.2 (−3.1, 3.4)	0.97
ΔKSS satisfaction	13.6 ± 8.4	16.3 ± 8.9	2.7 (−1.6, 7.0)	0.14
ΔKSS function	25.7 ± 23.2	30.3 ± 19.2	4.6 (−5.9, 15.1)	0.30
ΔKSS total	49.7 ± 35.8	56.4 ± 30.9	6.7 (−10, 23.4)	0.39

*Note*: Data are shown as the mean ± standard deviation.

Abbreviations: ΔaHKA, change in arithmetic hip–knee–ankle angle; CI, confidence interval; CPAK, coronal plane alignment of the knee; FJS‐12, Forgotten Joint Score; HKA, hip–knee–ankle angle; KSS, Knee Society Score 2011; PROM, patient‐reported outcome measure.

## DISCUSSION

The most important finding in this study was that postoperative lateral laxity was associated with larger changes in aHKA than with smaller aHKA changes in CPAK type I. However, the hypothesis that PROMs would be significantly worse in the larger ΔaHKA group than in the smaller aHKA group was not proved. Therefore, the concept that a large correction of constitutional coronal alignment would result in a large soft tissue imbalance was confirmed but its short‐term clinical influence was not. The rationale for under correction or maintenance of constitutional coronal alignment in restricted KA or adjusted MA remains controversial.

The clinical significance of this study is that MA TKA with large alignment corrections in aHKA results in residual postoperative lateral laxity, especially in CPAK type I with severe varus alignment. One of the reasons for this could be soft tissue laxity on the lateral side. Soft tissues, including ligaments that have sustained structural and cellular damage after reaching the threshold level of strain, are characterised by nonrecoverable changes in tissue length [29]. Soft tissues on the lateral side are laxer, especially in knees with severe varus deformity [9]. Colyn et al. considered that lateral joint laxity in the knee would be expected in severe varus and that such knees could show a mediolateral imbalance following TKA [3]. It was also reported that balancing the soft tissues during TKA would be more difficult in knees with severe varus deformity than in those with mild varus deformity [9, 26]. While constitutional varus deformities were not taken into account in previous studies, the new framework for CPAK classification will help surgeons decide on treatment strategy.

Although improvements in the FJS‐12, KSS satisfaction and KSS function scores tended to be better in the smaller ΔaHKA group (Table 3), the between‐group differences were not statistically significant. Nakahara et al. also found no correlation between PROMs and postoperative soft tissue laxity [23]. Therefore, ΔaHKA and soft tissue laxity do not appear to be strong independent factors that influence PROMs. Moreover, findings regarding improvement in PROMs were also comparable to previous studies [1, 2, 26].

One of the strengths of this study is that it evaluated changes in alignment based on the CPAK using both ΔaHKA and distance. Our findings suggest that a large alignment correction is not desirable in a knee with severe varus. However, the application of KA TKA in such a knee may lead to bone cuts with an overly oblique angle to the mechanical axis, and some authors have introduced restricted KA TKA with a safe zone [1]. Restricted KA sometimes needs correction of coronal alignment for knees with significant constitutional varus; therefore, postoperative lateral laxity would remain even in restricted KA although the correction angle would be smaller than that required with the MA method. The suitable limits for ligament balance and alignment [22] are still unclear, and further investigations are required.

Unlike classical coronal alignment using the HKA, JLO is a novel factor in the CPAK classification. This study did not investigate changes in mLDFA or MPTA independently because the coronal soft tissue balance would be influenced by the aHKA rather than the JLO. However, the clinical outcomes reflected in PROMs are influenced by many factors, which may include the mLDFA or MPTA [24]. Indeed, the philosophy of KA is to preserve the femoral geometry after TKA. The Journey II system was used in the present study, where the postoperative JLO would be 174° when performing MA and the postoperative distribution of JLO would be similar to the preoperative distribution; however, individual changes in mLDFA or MPTA after TKA may vary. There were no significant differences in the improvement of PROMs following MA TKA when focusing on the magnitude of alignment correction.

The CPAK classification does not take the joint line convergence angle (JLCA) into account. MacDessi et al. mentioned that they disregard the JLCA because it is approximately −0.5° in the normal knee, and its contribution to the prediction of constitutional knee alignment has minimal clinical significance [19]. JLCA is a metric for loaded extension gap balance [21]. The JLCA can be disregarded in the planning and assessment of TKA if the thickness of the polyethylene insert is symmetrical and there is no lift‐off of the femoral condyle from the tibial surface. When using an asymmetrical insert like the Journey II, the geometry of the insert should be considered. However, condylar lift‐off can occur if the residual lateral laxity is significant, especially when a large aHKA correction is needed. In a computer simulation study, Kuriyama et al. found that lateral laxity did not cause condylar lift‐off if the HKA angle was neutral but did if it was varus [16]. However, they manipulated the femoral alignment to create varus and did not consider the JLO. Therefore, the postoperative coronal alignment that should be aimed for knees with significant constitutional varus remains controversial: that is, (1) KA with no change in the aHKA and native soft tissue balance, (2) adjusted alignment, such as restricted KA with mild varus alignment and mild residual lateral laxity or (3) MA with neutral alignment and significant lateral laxity. Further research is needed to investigate the influence of detailed coronal alignment on CPAK and residual lateral laxity on the occurrence of condylar lift‐off in vivo.

This study had several limitations. First, bias could arise from the retrospective design. Implant selection was considered one of the major biases. Second, the sample size was relatively small. Third, the study was performed in an exclusively Japanese population. Constitutional varus alignment is known to be more common in Asian populations than in Western populations [18, 28, 33]. Fourth, as mentioned above, individual changes in mLDFA, MPTA and joint lines were not assessed. Given that MA TKA was performed in all patients, it is unclear whether KA TKA is an appropriate alternative. Furthermore, modifications, such as restricted KA, inverse KA and adjusted MA [1, 22], were not assessed. However, these modified alignment techniques require some amount of correction of aHKA; therefore, the present findings may be useful. Fifth, the flexion gap was not assessed. It is known that the flexion gap following TKA is a reliable indicator of soft tissue balance and is related to patient satisfaction and PROMs [12, 32]. Many factors influence PROMs, and Corbett et al. have suggested that it is not necessary to extend the CPAK classification beyond coronal plane alignment [4]. In addition, another issue is that methods for evaluating residual laxity have not been established and vary from literature to literature [7, 12, 14, 21]. Finally, the use of cruciate‐retaining inserts or bicruciate stabilised inserts in this study may have affected coronal laxity postoperatively. However, while significantly higher varus laxity in flexion has been reported with posterior stabilised TKA compared with cruciate‐retaining TKA, no such significant difference was found in extension [7].

## CONCLUSIONS

In this study, lateral laxity after MA TKA in knees with CPAK type I was more likely to be associated with larger changes in aHKA than with smaller changes in aHKA. However, no statistically significant differences in PROMs were found according to the amount of change in aHKA.

## AUTHOR CONTRIBUTIONS

All authors contributed to the study conception and design. Material preparation, data collection and analysis were performed by Junya Itou and Umito Kuwashima. The first draft of the manuscript was written by Junya Itou, and all authors commented on previous versions of the manuscript. All authors read and approved the final manuscript.

## CONFLICT OF INTEREST STATEMENT

The authors declare no conflict of interest.

## ETHICS STATEMENT

The study protocol was approved by the institutional ethics committee of Tokyo Women's Medical University (approval number: 4952). Informed consent was obtained via the opt‐out route.

## Data Availability

The data sets used and/or analysed during the current study are available from the corresponding author upon reasonable request.
